# ‘Compromise’ in Echolocation Calls between Different Colonies of the Intermediate Leaf-Nosed Bat (*Hipposideros larvatus*)

**DOI:** 10.1371/journal.pone.0151382

**Published:** 2016-03-30

**Authors:** Yi Chen, Qi Liu, Qianqian Su, Yunxiao Sun, Xingwen Peng, Xiangyang He, Libiao Zhang

**Affiliations:** 1 Guangdong Entomological Institute, Guangzhou, China; 2 Guangdong Public Laboratory of Wild Animal Conservation and Utilization, Guangzhou, China; 3 Guangdong Key Laboratory of Integrated Pest Management in Agriculture, Guangzhou, China; Texas A&M University, UNITED STATES

## Abstract

Each animal population has its own acoustic signature which facilitates identification, communication and reproduction. The sonar signals of bats can convey social information, such as species identity and contextual information. The goal of this study was to determine whether bats adjust their echolocation call structures to mutually recognize and communicate when they encounter the bats from different colonies. We used the intermediate leaf-nosed bats (*Hipposideros larvatus*) as a case study to investigate the variations of echolocation calls when bats from one colony were introduced singly into the home cage of a new colony or two bats from different colonies were cohabitated together for one month. Our experiments showed that the single bat individual altered its peak frequency of echolocation calls to approach the call of new colony members and two bats from different colonies adjusted their call frequencies toward each other to a similar frequency after being chronically cohabitated. These results indicate that the ‘compromise’ in echolocation calls might be used to ensure effective mutual communication among bats.

## Introduction

Communication behavior of animals is essential for the identification (including individual or population identification, and social class identification), breeding, formation, and maintenance of social relationships. Auditory communication may be employed over a range of distances and is particularly useful when vision is impaired [1]. Animals always possess their private acoustic signature, and their auditory systems appear to be specially attuned for hearing conspecific acoustical signals [2].

Calls are extremely important for survival of the bats that belong to the order Chiroptera and consist of approximately 1200 living species [3]. Seventeen of the eighteen chiropteran families are microchiropteran, which possess the ability to laryngeal echolocate, and this ability allows them to engage many activities that other vertebrates perform visually [4,5]. Echolocating bats collect information of their surroundings based on the differences between what they say and what they hear, and a higher frequency sound (shorter wavelength) is beneficial to maximize the details that they can locate their targets [6]. Echolocation signals of bats can be frequency-modulation (FM), constant-frequency (CF), or a combination of FM and CF syllable [7].

Because echolocation signals closely match the acoustic demands of echolocation, they would seem ill-suited for communication that requires variation to convey information [4]. Not surprisingly, non-sonar sounds (social calls), which differ from echolocation calls in some combination of duration, frequency, and pattern of frequency, change over time [8] and are used in a significant amount of communication among microchiropterans. Social calls that often have lower frequencies than echolocation calls [9] minimize the impact of attenuation and maximize the distance over which a signal can be heard [10]. Echolocation calls may also be used for social purposes, although social calls are the primary signals used in communication. [11,12,13,14,15]. Despite some studies focused on the communication role of FM syllables, there is little knowledge about the social function of CF echolocation calls.

Previous studies have shown that various social calls are often species-, group-, and even individual-specific, and each individual or species has unique acoustic parameters [16,17]. Social calls traditionally thought to encode a great deal of information about the caller allow individual recognition or group member recognition and help adult bats to relocate group members after periods of separation and maintain group cohesion [14,18]. Boughman found that call convergence occurred in certain bat populations: bats from one colony would converge with residents in screech calls after being transferred into the other bat colony that they joined [19]. Thus, colony-calls facilitate a communication among bats and coordinate bat activities. Nevertheless, bat audition is typically most sensitive to the frequencies used in echolocation [20]. Accompanied with social calls, bat echolocation pulses have been shown to be individual- and colony-specific, which might facilitate a bat’s ability to recognize individual and familiar groupmates [4,14]. Previous study exhibited the variation in the resting frequency of the constant-frequency component of bat calls when new bats converged with those of the original colony members [21]. However, it is unclear whether bats change their echolocation calls in free-ranging context, after living with a new colony, most notably, the mutual interactions of two colonies need to be elucidated. A more detailed understanding of how echolocation calls are involved in communication will allow better investigation of how ambient context influences their vocal behavior and information transfer within individuals or populations.

**Fig 1:**
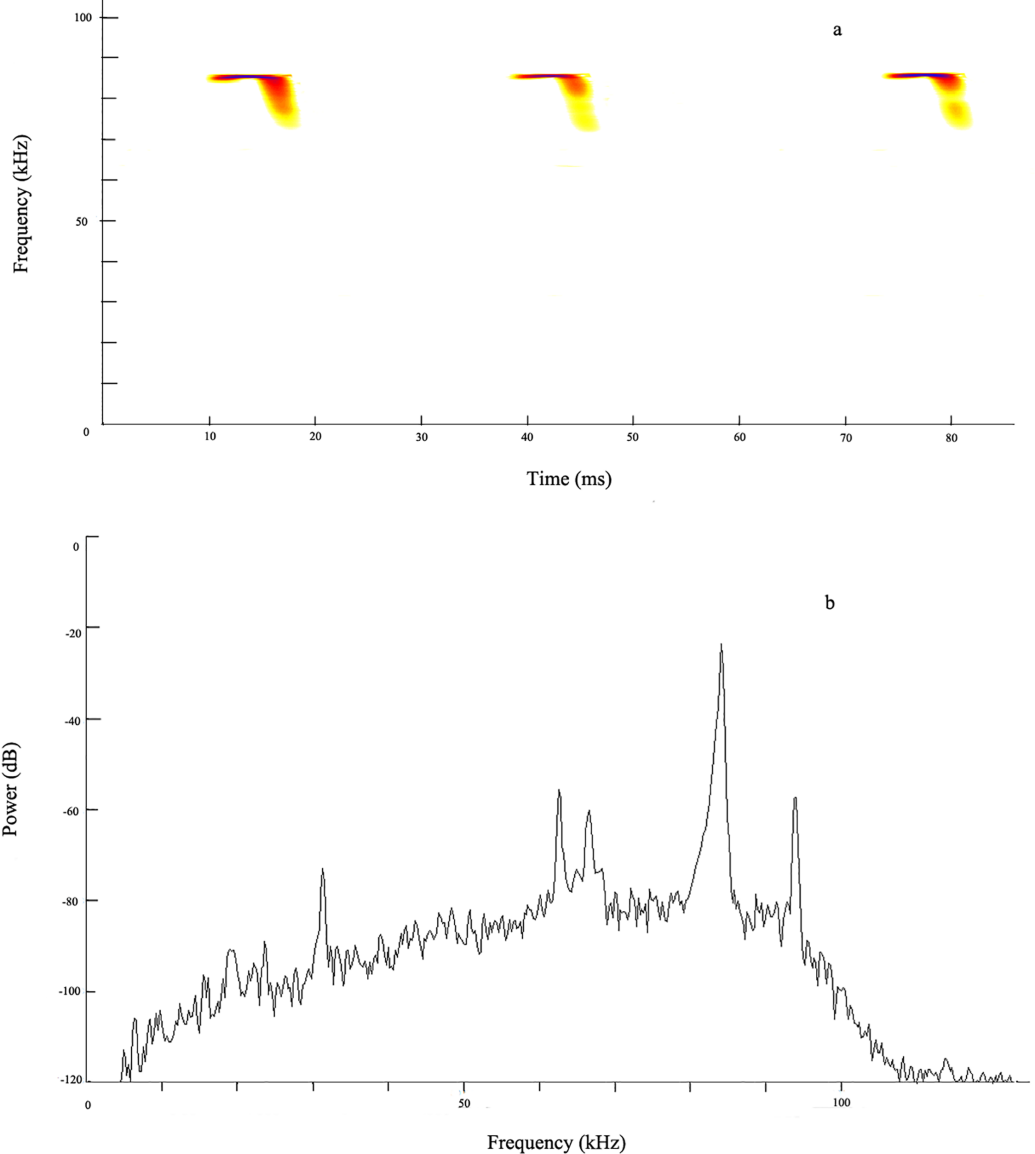
Sound spectrograms and power spectrum of echolocation calls of *Hipposideros larvatus*. a: sound spectrograms of echolocation calls; b: power spectrum of echolocation calls.

In the present study, the echolocation behavior of the intermediate leaf-nosed bat (*Hipposideros larvatus*), which is a widespread bat species in the Indo-Malay region (located in Asia with a geographical distribution that includes Bangladesh, China, Indonesia, Malaysia, Myanmar, and north-east India) [22], was investigated while the bats from different colonies converged. The echolocation calls of *H*. *larvatus* comprise a CF component that is an important factor to consider when characterizing echolocation strategy in CF-FM bats, followed by a short FM component (Fig 1). Evidence has been obtained to show that the divergence of *H*. *larvatus* in call frequency (ranging from 80 to 100 kHz in peak frequency) occurs in allopatry, and this acoustic character displacement is possibly caused by secondary contact to facilitate communication among bats from the same colony, rather than reflect phylogenetic relationships [23]. Acoustic convergence within social groups and differences between groups arise through vocal learning [19], which may coordinate group movements and facilitate mate recognition, and we hypothesized here that social environment may influence acoustic structure of echolocation calls. Bat acoustic signals show individual differences for jamming avoidance during fly [24]; however, group-specific in acoustic characteristics for communication is the premise of group living [20]. Furthermore, jamming avoidance is just a temporary change when bats are flying with other individuals [25], and bats should gradually adapting its pulse acoustics after joining a new group for identification and communication. We thus made the following predictions: (1) single bats should alter their echolocation calls to approach resident calls after entering a new colony and (2) two bats from different colonies cohabitate for a period of time would approximate echolocation calls of each other, by which they facilitate and ensure their mutual communication.

## Material and Methods

### Subjects

Twelve intermediate leaf-nosed bats were captured by mist net from two colonies in Shaoguan City (*A*: 3 males, 3 females), Guangdong Province (24°46.5′ N, 112°49.9′ E) and Guilin City (*B*: 3 males, 3females), Guangxi Province (25°16.7′ N, 111°21.0′ E), P.R. China. The peak frequencies of Colony *A* and *B* were 86.43 ± 0.25 (n = 11) and 82.84 ± 0.31 (n = 19) at the roosting area, respectively. Bats were individually placed in cloth bags and transported by car to Guangdong Entomological Institute, Guangzhou City, Guangdong Province, P.R. China (23°5.7′ N, 113°17.4′ E), where the experiments were performed. The two bat colonies were kept in cages (40 × 30 cm and 40 cm high) in separate rooms (3.5 × 3.5 m and 3 m high) where the environmental temperature was maintained at 24 to 28°C, and they can not listen to each other. Mealworms reared on a vitamin-enriched medium and water were always available *ad libitum*. We conducted the experiments from September 2012 to November 2013. All of the bats were returned to the capture sites and released after the experiments were completed.

### Experimental procedures

All of the methods were approved by the Guangdong Entomological Institute Animal Care Committee and were consistent with the ASAB/ABS Guidelines for the treatment of animals in research. Prior to testing, bats from different colonies were kept in separate rooms for one month, ensuring that bats habituated the experimental context and echolocation calls of bats remained stable in their own colonies [26].

#### Experiment 1: Do bats alter their echolocation calls to approach the resident calls after entering a new colony?

Bats from colony *B* (n = 6) were transferred singly into the home cage of colony *A* for one month. They were then separated from the residents and lived alone in a single cage for one month. The echolocation calls of all of the bats were recorded during the initial, transferred, and separated phases. In order to reduce the influence by co-adaptation of the two colonies, which may increase familiarity and weaken the differences of their initial signals, bats from colony *A* were not introduced singly into the home cage of colony *B* for the rest of experiments.

#### Experiment 2: Do different colony bats cohabiting for a period of time approximate to the echolocation calls of each other?

Two bats from different colonies were paired in one cage and left together for one month, and then, they were separated and placed alone in a single cage for one month. The echolocation calls of bats were also recorded during the initial, paired, and separated phases.

#### Sound recording and analysis

All of the calls were recorded when bats were free-flying in the laboratory. Echolocation calls were detected with an Avisoft Bioacoustics USG 116(e) detector (Avisoft Bioacoustics, Berlin) equipped with an Avisoft FG series microphone on a 2 m cable. The microphone was placed in the center of the test arena toward the flying bats. The USG 116(e) detector was connected to a laptop, and sound data were directly stored in the computer. The calls were recorded at a sampling rate of 250 kHz. Avisoft-SASLab Pro software (Avisoft Bioacoustics, Berlin) was used to analyze the parameters of the calls. Call parameters were measured automatically from spectrograms generated by consecutive 512 pt. FFT and 96.87% frequency overlapped with a Hamming window. Considering that there were no significant differences in the other call parameters (pulse duration, interval, bandwidth, and duty cycle) between the two colonies, we only measured the peak frequency from the spectra. All of the values were determined based on the second harmonic CF component (CF2).

#### Statistical analysis

We arbitrarily selected 50 pulses of echolocation calls (excluding those with a poor signal-to-noise ratio) for each individual in each trial and used the mean values of these 50 pulses for analysis. The statistical analyses were conducted using SPSS 16.0 for Windows. The normality and homogeneity of the variances were examined before analysis.

We compared the differences of their peak frequencies between the two colonies using a two-tailed t-test for independent-samples. A Bonferoni correction of repeated-measures was then used in multiple comparisons to analyze the alterations of the echolocation calls across the experiments. The statistical significance was set at p < 0.05. The measured parameters were expressed as the mean ± SD.

## Results

The peak frequencies of colony *A* (87.22 ± 0.29 kHz) and *B* (83.05 ± 0.27 kHz) had significant differences in the initial context before experiment 1 and 2 (independent-samples t-test: *t* = 25.463, p < 0.001).

**Fig 2:**
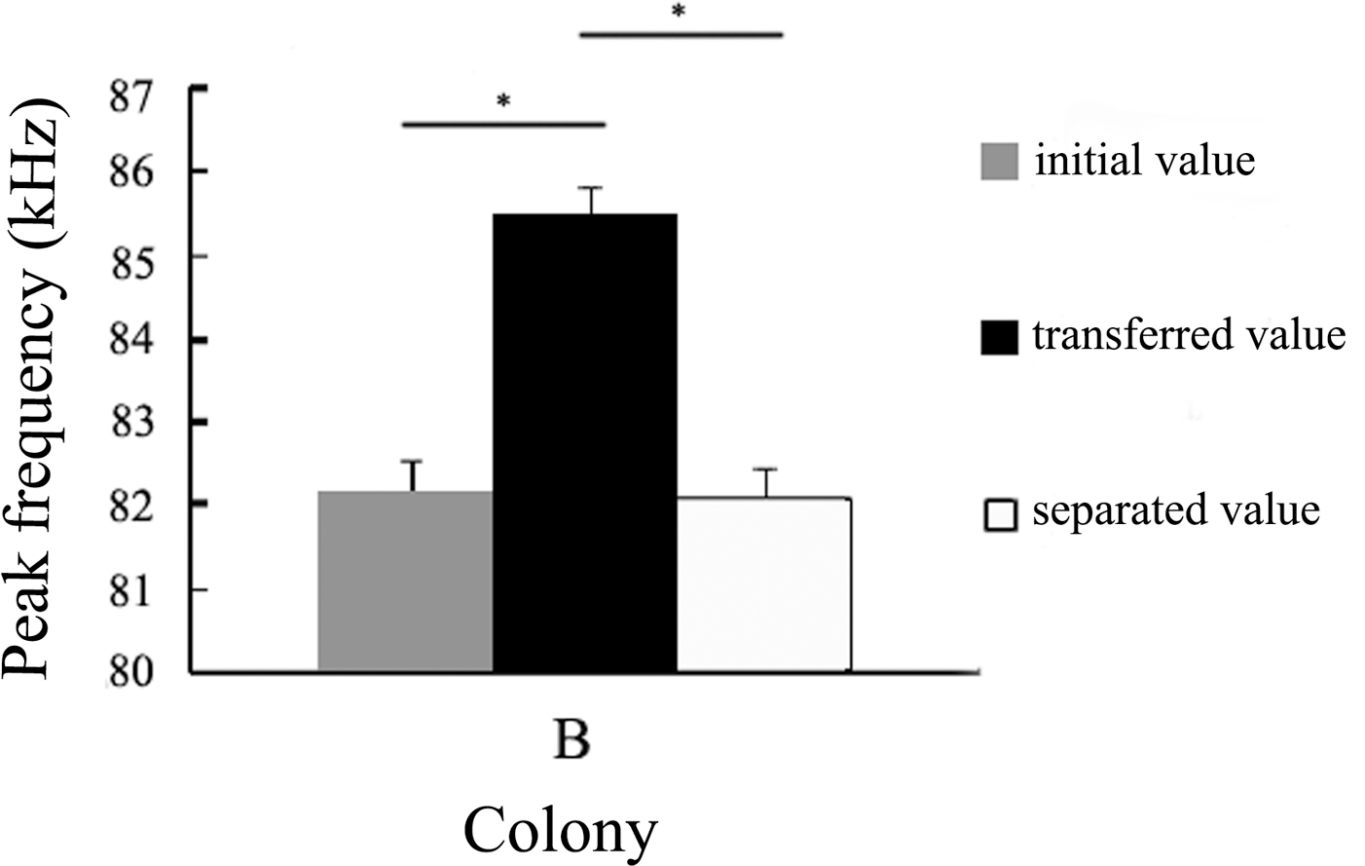
The variation of peak frequency of colony *B* bats when they were transferred singly into colony *A* (mean ± SD, n = 6). The asterisks indicate significant differences (medians and 95% credible intervals, Bonferoni correction).

In experiment 1, each of the individual bats from colony *B* that initially emitted echolocation calls with a lower peak frequency relative to colony *A* bats significantly increased its peak frequency after being transplanted into colony *A* for one month (n = 6, p < 0.001, Bonferoni corrected). However, the peak frequency decreased significantly after separation for one month (n = 6, p < 0.001, Bonferoni corrected), and the separated value did not show any difference from the initial value (Fig 2, Table A in S1 File). Additionally, the peak frequency of colony *A* remained stable across the entire experiment (all p-values > 0.05, Table B in S1 File).

**Fig 3:**
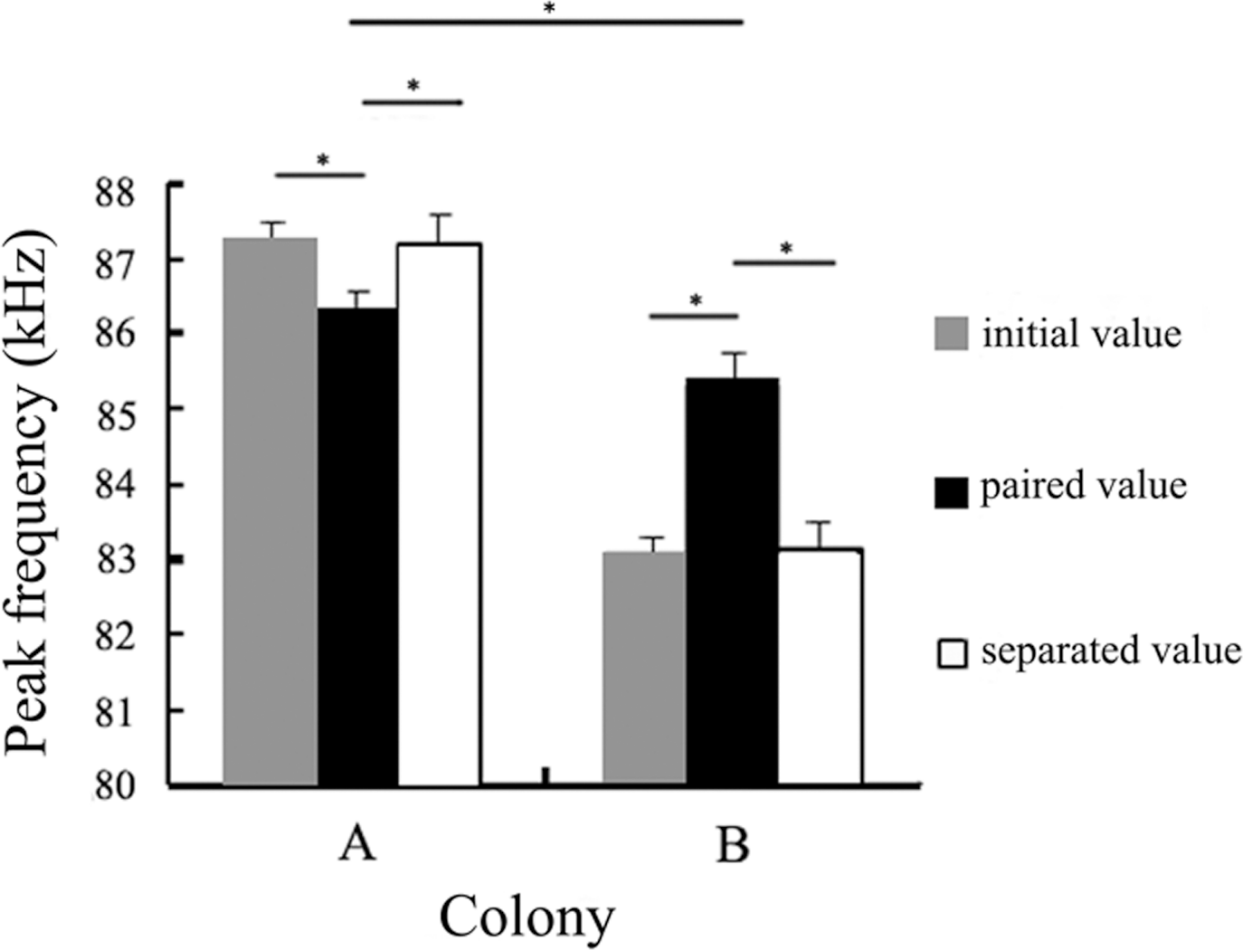
The variation of peak frequency of *A* and *B* colonies in the paired experiment (mean ± SD, n_1_ = n_2_ = 6). The asterisks indicate significant differences (medians and 95% credible intervals). A Bonferoni correction was used in the intra-group variation, and an independent-samples t-test was used to test the differences between groups. Paired condition: A_1_-B_1_, A_2_-B_2_, A_3_-B_3_, A_4_-B_4_, A_5_-B_5_, A_6_-B_6_.

In experiment 2, the peak frequencies of two colonies both changed significantly after being paired for one month; colony *A*, which had a higher initial value, decreased (n = 6, p < 0.001, Bonferoni corrected), whereas colony *B*, which had a lower initial value, increased (n = 6, p < 0.001, Bonferoni corrected). However, their call frequencies did not be totally converged, and their peak frequencies remained significantly different after being paired for one month (independent-samples t-test: *t* = 4.535, p < 0.01). Additionally, their peak frequencies were reversed after separation for one month; colony *A* showed an increase (n = 6, p < 0.001, Bonferoni corrected), whereas colony *B* decreased (n = 6, p < 0.001, Bonferoni corrected). Likewise, their separated values showed no significant difference with the initial values (Fig 3, Table C in S1 File).

## Discussion

Studies have demonstrated that bat echolocation calls show conspicuous plasticity. Variability in echolocation calls has been observed as a consequence of a variety of conditions, including searching or approaching prey [6,27] and recognizing complex surrounding [28,29,30,31]. Habitat segregation might play a role in preserving distinct echolocation calls at different geographical sites. Our results indicate that echolocation calls of *H*. *larvatus* possess high plasticity, lending support to previous studies that bats alter their echolocation calls in relation to their surroundings and permit the task that they are performing to function efficiently under different contexts [26,29,32,33]. Our findings offer strong evidence that bats adjusted their echolocation call structures to that of the residents after a single individual entered a new colony. Moreover, a ‘compromise’ phenomenon occurs in bat communication, and echolocation calls of different colony bats were altered and approximated to each other in the course of mutual communication. Our results support the hypothesis that social environment can give rise to the alteration of bats echolocation calls, and communication play a role in shaping the frequency bands utilized by bat communities.

Bats altered their call frequency to approach resident calls after entering a new colony, this social modification of echolocation calls is consistent with predictions for social learning of screech calls [19]. Sonar signals, like social calls, convey social information, such as species identity [23,34], contextual information, or other communicating messages [4,14,35]. Previous finding suggests that little brown bats, *Myotis lucifigus*, eavesdrop on each other’s echolocation sounds to locate patches of food [36]. Additionally, the interference (jamming) function of echolocation calls occurs in intra-specific food competition through the disruption of a competitor’s senses [24], and a cooperative function may also exist in the foraging context [15]. All of those changes are rapid and temporary for a given task. In the present study, however, bats adjusted their echolocation calls to habituate the resident colony calls so that their communication was effective, which may reinforce relationships with other individuals and facilitate their incorporation into the new colony. This indicates that bats have colony-specific echolocation calls as well as colony-specific audible calls, and we believe this is a gradual adapting process. On the other hand, the residents did not change their peak frequency after living with a single intruder for a month. One explanation could be that the probability of developing heterospecific acoustic depends on the relative magnitude of exposure to con- and heterospecific acoustic [37]. When mixed vocal occurs, it is usually most pronounced in the species with the lowest population density.

We also found that *H*. *larvatus* from different colonies chronically cohabiting will approximate each other’s echolocation calls when they were living in pairs. In this case, alterations would facilitate and ensure mutual recognition and unambiguous communication. It's worth mentioning that their call frequencies still showed significant differences after being paired for one month, which indicates that bats may maintain their own group specificity as group signatures for mutual recognition in addition to comfortably communicating with each other. Otherwise, bats from different colonies may converge not long enough, causing the ‘compromise’ of their calls to not full develop.

To ensure unambiguous communication, social calls with different functions often differ in structure [9], and each structure is highly stereotyped within a species. However, Russo et al. found that the social call structure showed a strong similarity between species, whereas echolocation calls were markedly different [38]. In fact, the structure of echolocation calls is flexible and is modified by bats in flight to optimize performance for certain tasks, such as target detection, localization and classification, in different habitats [29,39,40,41]. Hence, echolocation calls containing social information are prone to show a distinction among colonies with habitat segregation. Such differences are effective in allowing unambiguous communication within colonies. In one bat community, unified social or echolocation calls offer advantages for both the signaler and the receiver. This would increase individual fitness when the call frequency of new bats converged with those of the original colony members, and the ‘compromise’ of call frequency in different colonies after a long-term clustering period would increase their communication performance.

Hiryu et al. reported that bats continually showed a long-term and gradual change in call frequency over several months or seasons or throughout their lifetime, but all of the bats in the colony increased or decreased their call frequency as a group in the same direction [21]. In the present study, only the single individual changed its call frequency or both of the colonies altered their call frequency to the midpoint reciprocally, changes that occur were not in parallel in multivariate space, which indicate that observed changes cannot be explained by seasonal variation or physical environment alone, and the social environment clearly influences acoustic structure of calls. Additionally, Hiryu et al. revealed the intra-individual variation in the resting frequency of the constant-frequency component of bat calls; the resting frequency of new bats converged with those of the original colony members [21]. Our study showed that the peak frequency of echolocation calls in the flight state also altered when different group members converged over a prolonged period. The short- and long-term changes in call frequency may be associated with vocal learning, which is caused by social interaction or communication within the colony members.

In conclusion, echolocation calls do convey social information, and call structure may be modified by social experience. Although the small size of the laboratory colony could not fully represent a true bat colony, our findings suggest that bats show ‘compromise’ of their echolocation calls in the course of social interactions to ensure effective communication. This is similar to an interesting analogy of area-specific languages (dialects interplay) in birds [42] or humans [43]. Furthermore, call frequency of different colony bats reverts to the initial value after separation occurs again, which reveals that these changes are impermanent and invertible. Bat echolocation calls are specific, and habitat segregation might play a role in preserving distinct signals. The between-group differences and within-group convergence of bats echolocation calls may not only result from shared genes. The echolocation calls of bats are related to a long-term interaction with the local environment and evolved through gradual adaptation [44]. Therefore, understanding what social information echolocation calls contain and the role of echolocation calls in social contact is very important to develop a better perception of the bat vocal repertoire.

## Supporting Information

S1 File Table A:Peak frequency (kHz) of colony *B* bats when they were transplaced singly into colony *A* (n = 6). Table B: Peak frequency (kHz) of colony *A* bats when they live with a single bat of colony *B* (n = 6). Table C: Peak frequency (kHz) of *A* and *B* colonies during the paired experiment (n_1_ = n_2_ = 6).
(DOC)
