# Supplementary material for: ‘Compromise’ in Echolocation Calls between Different Colonies of the Intermediate Leaf-Nosed Bat (Hipposideros larvatus)
Source: PLoS One. 2016 Mar 30;11(3):e0151382. doi: 10.1371/journal.pone.0151382 (PMC4814132; doi:10.1371/journal.pone.0151382)
Supplement: S1 File — Table A:Peak frequency (kHz) of colony B bats when they were transplaced singly into colony A (n = 6). Table B: Peak frequency (kHz) of colony A bats when they live with a single bat of colony B (n = 6). Table C: Peak frequency (kHz) of A and B colonies during the paired experiment (n1 = n2 = 6). (DOC) [file pone.0151382.s001.doc]

**Table A.** Peak frequency (kHz) of colony *B* bats when they were transplaced singly into colony *A* (n= 6).

| Individuals | Gender | Initial value | Transferred value | Separated value |
| --- | --- | --- | --- | --- |
| B_1_ | male | 82.8 | 87.0 | 82.7 |
| B_2_ | male | 83.4 | 86.5 | 83.3 |
| B_3_ | male | 83.0 | 86.2 | 83.1 |
| B_4_ | female | 83.8 | 86.7 | 83.6 |
| B_5_ | female | 83.1 | 86.0 | 83.0 |
| B_6_ | female | 82.7 | 86.3 | 82.9 |

**Table B.** Peak frequency (kHz) of colony *A* bats when they live with a single bat of colony *B* (n= 6).

| Individuals | A_1_ | A_2_ | A_3_ | A_4_ | A_5_ | A_6_ |
| --- | --- | --- | --- | --- | --- | --- |
| Initial value | 87.3 | 87.5 | 87 | 87.4 | 86.6 | 87.5 |
| Mixed value-1 | 87.1 | 87.3 | 87.2 | 87.4 | 86.8 | 87.4 |
| Separated value-1 | 87.3 | 87.4 | 87 | 87.5 | 86.8 | 87.6 |
| Mixed value-2 | 87 | 87.2 | 87.1 | 87.3 | 87 | 87.3 |
| Separated value-2 | 87.1 | 87.2 | 87.3 | 87.4 | 86.9 | 87.5 |
| Mixed value-3 | 86.8 | 87.3 | 87.2 | 87.2 | 87.1 | 87.4 |
| Separated value-3 | 86.9 | 87.5 | 87.2 | 87.4 | 87 | 87.3 |
| Mixed value-4 | 87 | 87.4 | 87.1 | 87.1 | 87.3 | 87.2 |
| Separated value-4 | 87.3 | 87.4 | 87.3 | 87.1 | 87.2 | 87.3 |
| Mixed value-5 | 87.2 | 87.2 | 87.3 | 87 | 87.1 | 87.4 |
| Separated value-5 | 87.2 | 87.3 | 87.5 | 87.1 | 87.2 | 87.6 |
| Mixed value-6 | 87.1 | 87 | 87.5 | 87.2 | 87 | 87.6 |
| Separated value-6 | 87.2 | 87.5 | 87.1 | 87.4 | 86.7 | 87.4 |

“Mixed value-1” means that the colony *A* bats were cohabitated with B_1_ for a month; “Mixed value-2” means that the colony *A* bats living with B_2_ for a month; …

“Separated value-1”means that the colony *A* bats were separated from B_1_ for a month; “Separated value-2”means that the colony *A* bats were separated from B_2_ for a month; …

**Table C.** Peak frequency (kHz) of *A* and *B* colonies during the paired experiment (n_1_ = n_2_ = 6).

| Individuals | Gender | Initial value | Paired value | Separated value |
| --- | --- | --- | --- | --- |
| A_1_ | male | 87.2 | 86.3 | 87.3 |
| A_2_ | male | 87.5 | 86.7 | 87.5 |
| A_3_ | male | 87.1 | 86.1 | 86.9 |
| A_4_ | female | 87.4 | 86.3 | 87.3 |
| A_5_ | female | 86.7 | 86.0 | 86.5 |
| A_6_ | female | 87.4 | 86.5 | 87.6 |
| B_1_ | male | 82.8 | 85.6 | 82.8 |
| B_2_ | male | 83.2 | 85.8 | 83.4 |
| B_3_ | male | 82.9 | 85.0 | 83.0 |
| B_4_ | female | 83.5 | 85.8 | 83.8 |
| B_5_ | female | 83.1 | 84.7 | 83.1 |
| B_6_ | female | 82.8 | 85.2 | 82.7 |

Paired condition: A_1_-B_1_, A_2_-B_2_, A_3_-B_3_, A_4_-B_4_, A_5_-B_5_, A_6_-B_6_.
